# Two Decades of Medical Education Scholarship: Mapping Collaboration and Thematic Shifts Using Web of Science (2000–2019)

**DOI:** 10.5334/pme.1853

**Published:** 2025-11-10

**Authors:** Anton Boudreau Ninkov, Constance Poitras, Jason R. Frank, Joseph Costello, Lauren A. Maggio, Anthony R. Artino

**Affiliations:** 1 Université de Montréal, École de bibliothéconomie et des sciences de l’information in Montréal, Québec Canada; 2 Department of Emergency Medicine and Centre for Innovation in Medical Education, University of Ottawa, CA; 3 Department of Medical Education, University of Illinois Chicago School of Medicine, US; 4 School of Medicine and Health Sciences, George Washington University, US

## Abstract

**Introduction:**

The field of medical education (ME) has grown substantially over the past decades, yet questions remain about its scope and boundaries. This study examines how research topics and institutional collaborations have evolved in ME from 2000 to 2019.

**Methods:**

Adopting a post-positivist stance and using bibliometric network analyses, we examined metadata from 31,338 publications across 22 core ME journals indexed in the Web of Science. We analyzed trends in institutional collaboration and the development of research themes. Extracted metadata included authors’ institutional affiliations and KeyWords Plus (n = 18,218). Bibliometric analyses were conducted using VOSviewer, a widely used tool for network mapping. We generated co-authorship networks to trace institutional collaboration and co-word networks to identify thematic clusters.

**Results:**

Co-authorship networks revealed increasing collaboration, with U.S. institutions remaining central and Canadian and Dutch institutions gaining prominence. Co-word analyses identified three stable clusters—teaching and learning, quantitative, and psychosocial—with teaching and learning dominant across all periods and the quantitative cluster expanding in recent years.

**Discussion:**

Findings show the consolidation of teaching and learning as the foundation of ME, alongside diversification through quantitative and psychosocial themes. Growing collaborations suggest the field’s maturation, though geographic imbalances persist. Limitations include reliance on a restricted set of Web of Science journals, which overrepresent English-language and highly cited publications, and the use of KeyWords Plus as a proxy for themes. This study offers an evidence-based mapping of ME’s evolution and provides a framework for future research on the interdisciplinary and global dynamics of the field.

## Introduction

Scholarly communication and collaboration between researchers [1] facilitate the dissemination of knowledge, methods, and techniques from one discipline to another, leading to the emergence of new interdisciplinary fields of research [2]. Research collaboration is crucial to creating and connecting knowledge and skills across different disciplines [3]. Collaboration takes many forms, including between individuals, institutions, specialties, and countries. The reasons for creating collaborative research networks are varied, such as combining resources, promoting knowledge advancement, capitalising on specialties, and addressing interdisciplinary research problems [4]. Research collaboration can expand the research topics of a discipline or field, particularly in international and cross-cultural collaborations [5]. Using bibliometrics to study the historical development of fields of knowledge is not a new approach [6]. Such studies are particularly relevant when they focus on emerging knowledge domains or domains at the intersection of multiple disciplines, such as nanoscience [7], climate change research [8], or the field of sustainability science [9].

Medical education (ME) has grown substantially over the past two decades, becoming an increasingly interdisciplinary and collaborative field. Researchers from diverse backgrounds, including educators, clinicians, psychologists, sociologists, and policymakers contribute to the development of the field, which now encompasses a wide range of methods and topics [6,7,10]. This diversity fosters innovation but also makes it difficult to define the boundaries of ME as a distinct scholarly domain [12,13]. Prior studies have used bibliometric approaches to explore specific aspects of ME but few have examined the field’s evolution over time. In particular, there is limited understanding of how research topics have shifted and how institutional collaborations have developed in parallel. Without this broader perspective, it is difficult to evaluate how ME has matured as a field, which areas have gained prominence, and how collaborations have contributed to knowledge production.

This study addresses this gap by mapping the evolution of research topics and institutional collaborations in ME from 2000 to 2019. By combining a historical analysis of research themes with patterns of collaboration, we provide a multifaceted understanding of the field’s development over two decades. The 20-year timeframe allows us to identify long-term trends while avoiding the confounding effects of the COVID-19 pandemic on publishing patterns. Insights from this analysis can help researchers, institutions, and policymakers reflect on the growth of ME, strengthen cross-disciplinary partnerships, and inform strategic decisions about the future direction of the field. Following Boyer’s framework [14], we situate our work within the scholarship of discovery, focusing on research activities that advance knowledge and explore new topics in ME. Therefore, this study addresses the following question: How have research topics and institutional collaborations evolved in the field of ME from 2000 to 2019?

## Background

To examine a field, it is important to first be able to adequately *define* it [7,15]. Defining a field involves identifying the most popular subjects, the institutions that are most central to the development of new themes, and even the preferred methods of dissemination [16]. Certain aspects of the field of ME have been previously explored and described. For example, between 1999 and 2019, there has been a general increase in the number of authors contributing to knowledge syntheses in ME, an important type of publication in this field [17]. Azer [18] also examined publication types and venues in ME and found that, among the 50 most cited publications in the field, 20 were published in general medicine, educational psychology, and higher education journals. He also highlighted that only eight of the most cited papers were research publications. Also, Rangel et al. [19] analyzed the top-cited papers in *Medical Education* and found that 80% of these publications were review papers, with empirical research appearing much less frequently. These findings suggest that ME places significant emphasis on knowledge syntheses and review publications.

Epistemic injustice in ME has also been studied, revealing that the Global North has dominated authorship in major ME journals, shaping perceptions of “who counts as an expert” and “whose knowledge matters.” [20]. This has consequences for the development of general educational theories and practices in ME, which have largely neglected to incorporate perspectives from marginalized racial groups [21]. Finally, the field’s interdisciplinary nature has been studied, and it has been observed that, to some extent, ME is inward-looking in that it heavily cites its own research [22].

Bibliometric methods and network analysis have been used previously to identify ME research topics. For example, keywords from 9379 publications between 1963 and 2015 and indexed in PubMed have been analysed to identify five historical phases in the development of the field of ME: the waking phase (1963–1975), the birth phase (1976–1990), the growth phase (1991–1996), the maturity phase (1997–2005), and the expansion phase (2006–2015). The first three phases are marked by the emergence of new keywords—an evolution that reflects efforts to address societal needs through educational paradigms in a field long dominated by a positivist approach—while the fourth and fifth phases are characterized by the formation and strengthening of networks [23].

Several studies have identified the main research topics in ME. Content analysis has been used: [16] where researchers conducted an extensive analysis of 10,168 abstracts from six journals in ME between 1988 and 2010. This study identified 29 major themes, the most prominent of which were student assessment, clinical and communication skills, clinical clerkships, and problem-based learning. More recently [24], content analysis has been used to examine 1126 studies published in five core ME journals at three time points (2000, 2010, and 2019). The authors identified several themes that remained consistently important across the three time points, such as effective teaching methods, clinical skills development, and curriculum fitness for purpose. Along the same lines, a study analyzed ME publications indexed in MEDLINE between 1960 and 2010 using medical subject headings and identified four main research topics: curriculum and teaching issues, professionalism, medical student characteristics, and assessment [25].

Interviews have also been used to examine this issue [26], with one notable example being a study involving 21 editorial board members representing nine of the top ME journals who identified what they perceived as the most important research topics in ME. This approach found that topics related to physician competencies (skills, knowledge, attributes), curricula, and teaching are central. Topics related to research methodology, program evaluation, and technology are also increasingly important. Furthermore, the use of sequential [27] mixed-methods study design, with a qualitative and quantitative survey followed by a qualitative focus group, to identify priority research topics in ME has been used. With this approach, the top three identified included: understanding how to ensure that students develop the skills needed for work, how to promote student resilience and well-being, and ensuring that the curriculum prepares students for work.

In the context of ME research, where collaboration is central [11], it is necessary to understand how collaboration between institutions is structured, as the latter may prove to be a mechanism by which the topics studied in ME evolve. Scientific collaboration involves interactions in a social context between at least two scholars, institutions, or countries to facilitate the sharing of meaning, and the accomplishment of tasks aligned with a common goal [28]. The motivations for establishing collaborative networks are numerous [29], and certain factors, such as geographic proximity, the sharing of a common language, as well as other social and political factors [30], can explain the network dynamics. Moreover, collaboration between institutions has generally allowed for the production of science with greater impact [31,32] and generally plays an important causal role in enabling scientific communities to realise their epistemic goals [33].

In ME, multi-institutional research is viewed in a positive light, as it increases the generalizability of research findings [34], and a few studies have examined collaboration between institutions or between countries. Sarkar et al. [24] found a gradual increase in multi-institutional studies in ME between 2000 and 2019, while noting the dominance of US institutions. Other studies have also noted the dominance of Western English-speaking countries in ME publications [18,19,35,36,37,38]. The current study expands this previous work.

## Methodology

The boundaries of a field can be defined in a number of ways. Field delineation is often seen as the first step in a research process, allowing scholars to explore the nature of a discipline. For this study, we adopted a post-positivist stance and used bibliometric methods which rely on metrics to characterize relationships between published research in a field. For this analysis, we chose to base our research on the Medical Education Journal List 24 (MEJ-24), which is a seed set of 24 journals [14] (see Appendix 1). We then collected metadata from the Web of Science (WoS) for all publications in 22 of the 24 journals between 2000 and 2019, resulting in a dataset of 34,768 publications, including citation data. The two journals for which data were not collected included the *Journal of Graduate Medical Education* and the *Canadian Medical Education Journal*, which were missing from the WoS. Using VOSviewer, we performed a co-citation analysis, identifying 66,833 instances where journals were cited together, mapping key intellectual relationships within the field. Additional information on the methods used to obtain the dataset has been previously described in further detail [13].

### Data collection and analysis

For this study, we chose to use the WoS as a data source because it provides well-defined metadata that we could use to create the networks shown here. The data used in this study were pulled during the fall of 2021, approximately 2 years after the 20-year period under analysis in this study. From our perspective, this is ample time for the appropriate data to have been added to the WoS database for a complete picture of this time period, which research has shown is achieved 9-months after the year’s completion [39].

For the data analysis, we chose VOSviewer, a freely available software tool designed for constructing and visualizing bibliometric networks [40]. We selected VOSviewer because it is particularly well suited for visualizing large networks of co-authorship and thematic clusters, which are central to the objectives of this study. VOSviewer has been widely used in bibliometric studies to map research topics and collaboration networks in diverse fields [41,42,43,44].

### Institutional collaborations

To establish a network of institutional collaborations, we extracted 31,338 papers published from the beginning of 2000 to the end of 2019 in MEJ-24 journals from WoS (20 years). For each paper, we extracted the authors’ affiliation and created a network where the nodes represent research institutions, and the edges represent collaborations within the same paper. To observe the research collaboration patterns, we took snapshots in 5-year periods from 2000 to 2019 (2000–2004, 2005–2009, 2010–2014, 2015–2019). This study used a thesaurus of institutions developed for a previous related study [45] to account for the various ways the same institution can be written, and applied that to the collaboration network analysis.

### KeyWords Plus

In an effort to see disciplinary boundaries in ME, we used KeyWords Plus from VOSViewer to examine what subjects were being discussed. To demonstrate the development of interdisciplinary themes in ME, we extracted the KeyWords Plus from publications in the MEJ-24 between 2000 and 2019, resulting in a total of 18,218 keywords. KeyWords Plus were used for our project owing to their extensive repertoire of terms and their broad meanings. This attribute notably accentuates the research methods and techniques presented within the publications and more effectively than the keywords provided by the authors [46].

To have the KeyWords Plus list better reflect the characteristics of the ME field, we created a thesaurus [47] based on the KeyWords Plus that had a minimum of 50 appearances in the listed time period (total of 816 words). The thesaurus was designed to merge only morphological variants of the same term (e.g., *teacher* and *teachers*), without collapsing distinct but related concepts (e.g., *education* and *teaching*). The merging was performed manually by one member of the team and independently validated by another to increase consistency and reduce potential bias. The complete datasets, including the thesaurus, have been openly published and are accessible for reuse, ensuring transparency and reproducibility of the analysis. We then used the 100 most frequently occurring KeyWords Plus to create a co-occurrence network for each 5-year period, which illustrated the evolution of the major trends in ME.

### Analysis of the bibliometric networks

The bibliometric networks presented in this paper were created using VOSViewer. With the dataset files pulled from WoS and stored locally, we used VOSViewer to analyse each of the 5-year periods based on co-occurrences of KeyWords Plus and collaborations of institutions. We chose these two analyses to reveal major research themes generated in this field (and potentially highlight the interdisciplinarity of the field) as well as reveal the global boundaries and groupings of researchers in the field. Specifically, for the keyword networks, analysis settings included: a minimum cluster size of 10; 0.90 resolution; the top 100 KeyWords Plus; and no lines to better see each word. For the institutional collaboration networks, analysis settings used included: a minimum cluster size of 10; 0.80 resolution; the top 100 institutions (by output); lines between nodes with a minimum strength of 5.

VOSviewer applies a modularity-based algorithm that groups nodes into clusters when they are more strongly connected to each other than to the rest of the network. Clusters therefore represent sets of institutions that collaborate more frequently, or sets of topics that are commonly studied together. To keep networks interpretable, we restricted each map to the top 100 institutions or keywords in a given period, and we applied minimum cluster sizes (10) and link-strength thresholds (5 for institutional collaborations). For co-citation analyses used in field delineation, a minimum of 50 co-citations was required. It is important to note that network maps should be read as heuristic tools where distance indicates relative relatedness, while cluster colour signals overall grouping. They do not imply hard disciplinary boundaries but instead highlight structural patterns and thematic tendencies.

## Results

We present our findings in two subsections: the network maps for institutional collaboration and the network maps for Keywords Plus, respectively. Using data from each of the five-year time periods specified (2000–2004, 2005–2009, 2010–2014, and 2015–2019), we present four network maps in each section, as well as discuss how the maps reflect changes in the field of ME over time.

**Figure 1:**
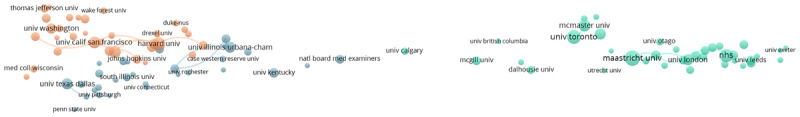
Institution collaborations network 2000–2004.

**Figure 2:**
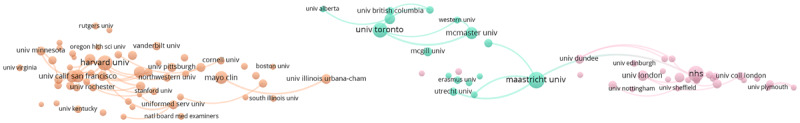
Institution collaborations network 2005–2009.

**Figure 3:**
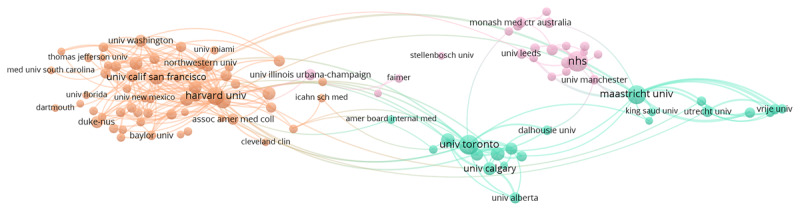
Institution collaborations network 2010–2014.

**Figure 4:**
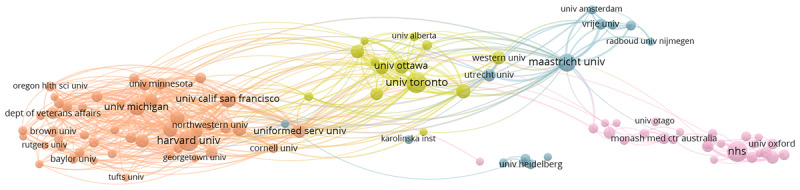
Institution collaborations network 2015–2019.

The network maps presented in this paper (Figures 1, 2, 3, 4) can be understood in the following way. Each map is made up of 100 data points (Institutions or Keywords Plus). These data points are plotted closer together if the metadata suggest they are similar and further apart if they are not. Their colour indicates what general cluster they belong to, meaning an overall similarity between the data points of similar color. When lines appear on the network map (Figure 1), this indicates that a strong connection between the data points is present.

### Institutions

Institutional collaborations in the field of ME evolved from 2000–2019 (Figures 1, 2, 3, 4). The number of clusters, representing closely linked groups of institutions, increased from 3 clusters in 2000–2004 to 5 clusters in 2014–2019. As well, the number of overall collaborations (links) between institutions analyzed increased over time with 855 links observed in 2000–2004 and 2029 links observed in 2015–2019. Although institutions, and not countries, are the unit of measurement in this section, clear regional relationships seem to emerge. In the earliest time period (Figure 1), there were three apparent clusters: 2 clusters stemming from the USA (red and dark blue) and then an international cluster including universities in Canada and across Europe (green). There was a clear divide where the 2 USA clusters were more connected, while the rest of the world was more separated. In 2005–2009 (Figure 2), three clusters emerged. However, this time the USA cluster merged into one (red), a Canadian/Dutch cluster (green) and an England/Australia cluster (pink) emerged. It is noteworthy that while the USA cluster is still apart from the rest, the Canadian/Dutch cluster sits between the two ends, with England and Australia on the other side. In 2010–2014 (Figure 3), once again the three clusters present in the 2005–2009 network emerged, but a fourth cluster separating Canadian and Dutch researchers was beginning to develop. This fourth cluster was actualized in the 2015–2019 network (Figure 4), where we see the USA cluster on the left (red), the Canadian cluster in the middle (yellow) but closer to the USA cluster, the Dutch cluster in the middle (light blue) but closer to the England/Australian cluster located on the far right (pink).

### KeyWords Plus

The networks of KeyWords Plus reveal several clusters in the four specified time periods from 2000–2019. Collectively, throughout all the time periods, 18,218 Keywords Plus were identified and three clusters were apparent. The first was teaching and learning (green), which consisted of indicating words such as: students, model, faculty development, instruction. The second cluster was the quantitative or positivist (blue), which consisted of words such as: checklist, costs, performance assessment, and competency. Finally, there was also a psychosocial cluster (red), which consisted of words such as: attitude, behaviour, stress, and depression.

**Figure 5:**
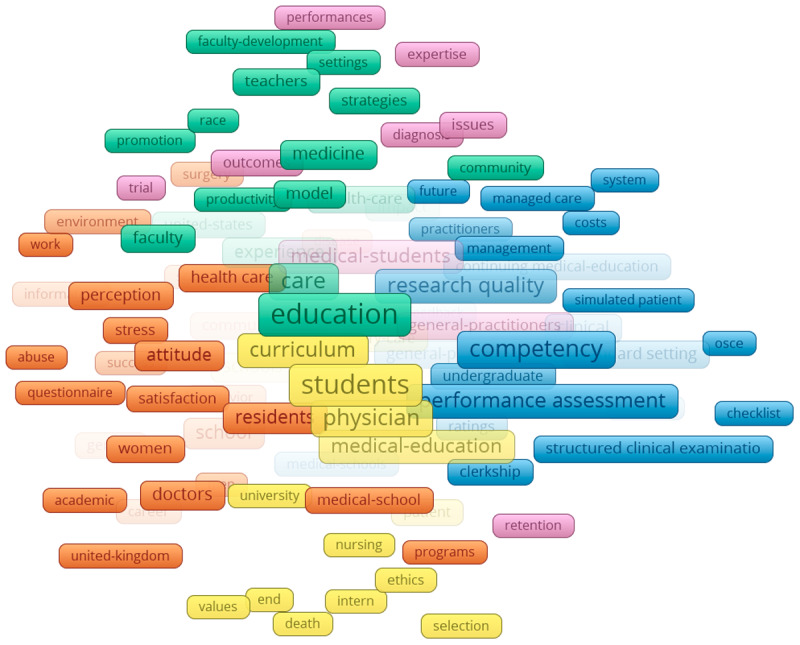
KeyWords Plus network 2000–2004.

**Figure 6:**
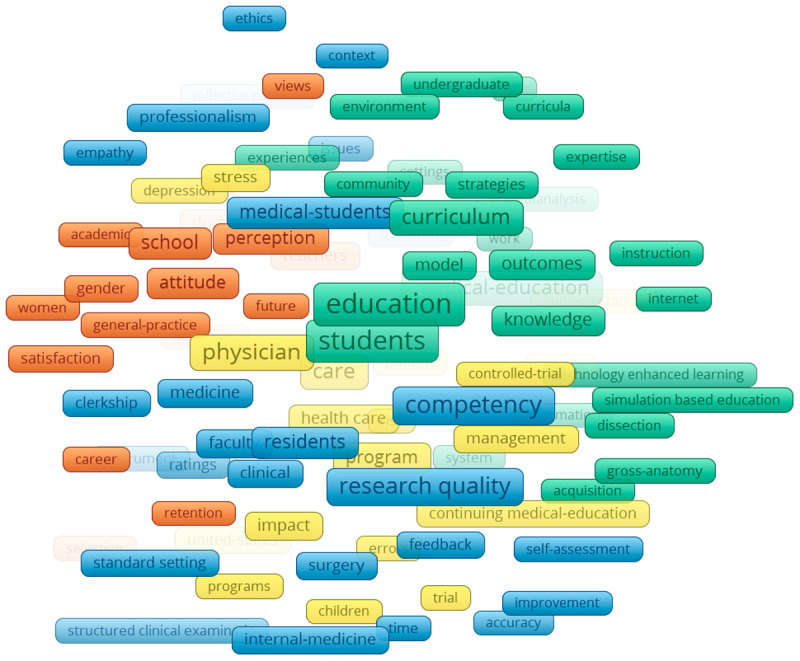
KeyWords Plus network 2005–2009.

**Figure 7:**
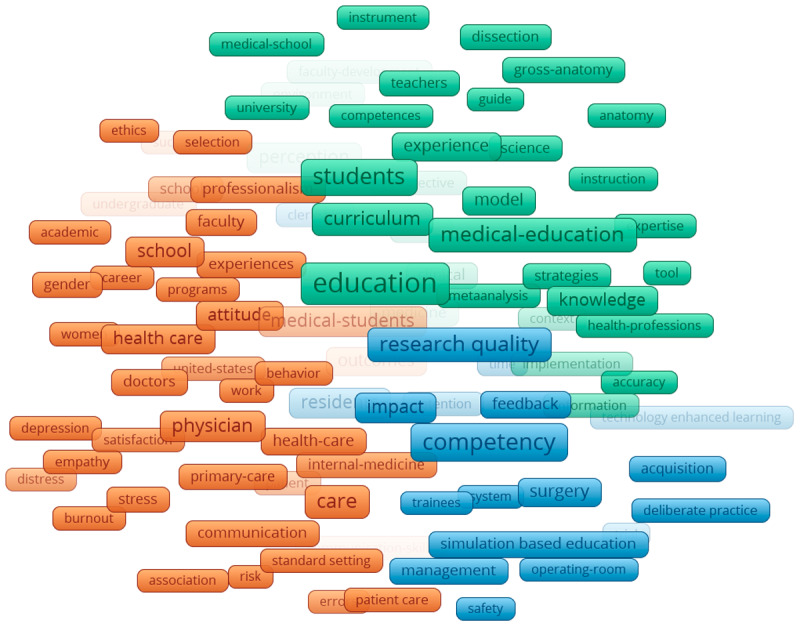
KeyWords Plus network 2010–2014.

**Figure 8:**
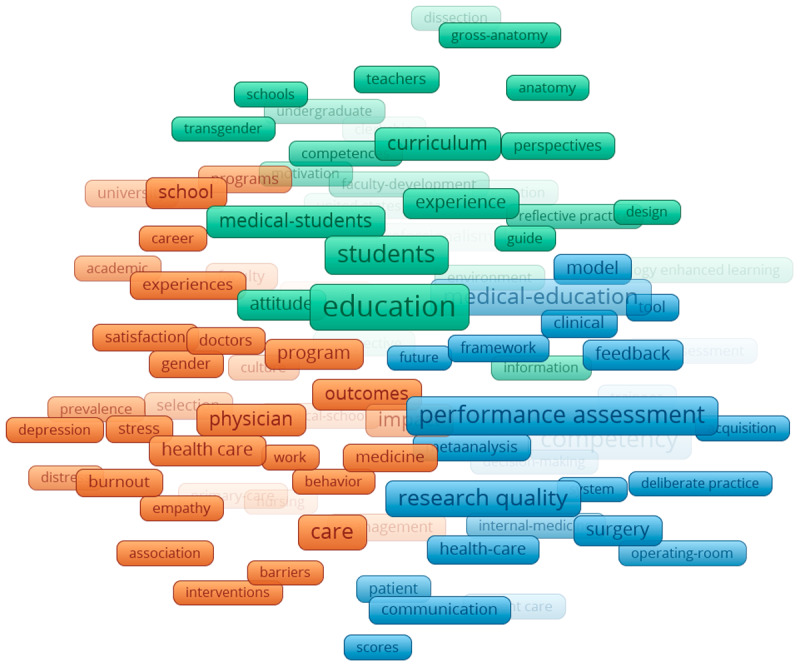
KeyWords Plus network 2015–2019.

Specifically, in the earliest time period (Figures 5), two clusters appear to be mostly related to teaching and learning clusters (pink and yellow). The teaching and learning cluster spanned the middle of the network, with the other two clusters on separate sides. In the 2004–2009 time period (Figure 6), four clusters appeared and, similarly to the earliest period cluster, teaching and learning encompassed multiple clusters and spanned mostly in the middle. In the last two time periods (Figures 7 and 8), the three main groupings emerged and are clearly divided. The biggest change between the two later time periods is that the quantitative/positivist cluster grew.

## Discussion

From the network maps of institutional collaborations and KeyWords Plus, patterns emerged that provide a more complete understanding of the evolution of the field of ME over a 20-year period. It is important to note that this study was designed as an exploratory, descriptive analysis of structural patterns and thematic trends. Although the results could be examined through various sociological or theoretical lenses, our aim here is to provide a foundational mapping of the field that future research may interrogate using specific theoretical frameworks. Below, we discuss our findings, including limitations and future work.

### Institutional collaborations

A major takeaway from our findings is that overall, collaborations are increasing, corroborating previous work showing that collaboration helps the scientific community achieve epistemic goals and explains its persistence and growth [33]. Factors favouring collaboration, such as sharing a common language and culture [30], are also highlighted in our results, with the first collaborations occurring between the United States, Canada, and the United Kingdom. Noticeably absent from our collaborative networks is the Global South, confirming the findings of Maggio et al. [20] and Guragain et al. [38] that this region has been largely absent from the scholarly discourse. Also, the dominance of institutions from Western English-speaking countries, as identified by several authors [24,35,36,37], is evident in our results, with the exception of the Netherlands. An additional source of increased institutional collaboration may be the rapid growth and expansion of graduate programs in ME, many of which attract a global student body [48].

### Interdisciplinarity as seen through KeyWords Plus

The keyword analysis provides a visual representation of the interdisciplinarity of the field of ME. The generated network maps show the research topics related to medical sciences coexisting along with social sciences, education, and psychology. Our main keyword finding is the consolidation of topics over time. As the time periods progress, the teaching and learning, quantitative, and psychosocial clusters consolidate, further developing the findings of Ji et al. [23] and Rotgans [16] who showed the expansion of research themes related to psychosocial issues.

The findings of this study align with and extend prior research on the evolution of research topics in ME. Consistent with earlier studies that used content analysis (e.g., [16,24], the results highlight teaching and learning as a central and enduring theme in ME research. The dominance of this cluster across all four time periods (2000–2019) and its position at the centre of the network corroborates earlier findings that topics such as effective teaching methods and curriculum development are core to ME [24,26]. Furthermore, the persistence and growth of the quantitative/positivist and psychosocial clusters suggest an increasing diversification of research approaches and themes over time.

The quantitative/positivist cluster, characterized by terms such as “performance assessment” and “competency,” reflects the emphasis on measurable outcomes and competencies. The growth of this cluster over time may also reflect the broader shift in ME research toward data-driven methodologies. Additionally, the psychosocial cluster aligns with Palermo et al.’s [27] findings that student resilience, well-being, and attitudinal development are emerging priorities in ME research. The presence of terms such as “attitude,” “behaviour,” and “stress” within this cluster highlights a growing interest in addressing the psychosocial aspects of medical training, a topic that has gained prominence in recent years.

The evolution of clusters across time periods also reveals interesting patterns. In the earliest period (2000–2004), the presence of five clusters, including two additional teaching-related groupings, suggests a broader focus on teaching and learning topics during this phase. Over time, these clusters consolidated into the three distinct groupings observed in the later time periods. This consolidation mirrors the findings of Ji et al. [23], who noted a narrowing of focus to core themes during the maturity and expansion phases. The central position of teaching and learning throughout all time periods underscores its foundational role in ME.

### Limitations and Future Work

In terms of limitations, the research sample was derived from the MEJ-24, an initial set of journals that does not necessarily encompass all publications relevant to ME. Moreover, because the MEJ-24 was drawn from the WoS, the sample inherits the database’s limitations: WoS indexes only a selective subset of the most cited journals, with a strong overrepresentation of English-language publications. As a result, research outputs such as articles in highly specialized or national journals, book chapters, preprints, and work published in languages other than English are likely to be underrepresented, which may introduce biases in the coverage of the field [49,50]. Additionally, WoS does not cover 2 of the 24 journals, further limiting the scope of the dataset we acquired. Finally, the thesaurus used for the networks generated in this study lacks completeness as we completed it for a subset of all the KeyWords Plus obtained previously. The reliance on KeyWords Plus as a basis for network analysis may exclude important nuances captured in full-text analysis or other methods. Future research could integrate qualitative approaches or stakeholder perspectives to further validate and refine these findings. Future analyses using more recent data may help contextualize our historical findings and identify post-COVID differences in collaborations between ME researchers.

## Conclusions

This investigation into the evolution of ME sheds additional light on the internationality and interdisciplinary nature of the field. Through the network maps, we were able to visualize these complex patterns, revealing the increasing global interconnectedness of institutions and the blending of medical, social, and psychological sciences to address complex educational challenges. These findings contribute to the discourse on field evolution by highlighting the thematic consolidation within ME research over two decades, with teaching and learning remaining a foundational theme. While important limitations exist, this study of ME not only enhances our understanding of the field’s historical trajectory but also establishes a methodological framework for examining evolutionary patterns within other fields and disciplines.

## Appendix 1- MEJ-24 Journal List

**Table:**

Academic Medicine

Medical Education

Medical Teacher

Anatomical Sciences Education

BMC Medical Education

Advances in Health Sciences Education

Teaching and Learning in Medicine

Journal of Continuing Education in the Health Professions

Journal of Surgical Education

Journal of Graduate Medical Education

Clinical Teacher

Medical Education Online

GMS Journal for Medical Education

Simulation in Healthcare

Advances in Medical Education and Practice

Education for Health

Perspectives on Medical Education

International Journal of Medical Education

Journal of Educational Evaluation for Health Professions

African Journal of Health Professions Education

Journal of Medical Education and Curricular Development

Canadian Medical Education Journal

Focus on Health Professional Education

BMJ Simulation & Technology Enhanced Learning
